# Draft Genome Sequence of *Bacillus muralis* LMG 20238^T^ (DSM 16288), a Spore-Forming Bacterium Isolated from Deteriorated Mural Paintings

**DOI:** 10.1128/genomeA.01691-15

**Published:** 2016-02-04

**Authors:** Jie-ping Wang, Bo Liu, Guo-hong Liu, Qianqian Chen, Zhizhen Pan, Xue-fang Zheng, Meichun Chen

**Affiliations:** Agricultural Bio-Resources Research Institute, Fujian Academy of Agricultural Sciences, Fuzhou, Fujian, China

## Abstract

*Bacillus muralis* LMG 20238^T^ is a Gram-positive, aerobic, and spore-forming bacterium. Here, we report the 5.18-Mb draft genome sequence of *B. muralis* LMG 20238^T^, which is the first genome sequence of this species and will promote its fundamental research.

## GENOME ANNOUNCEMENT

The type strain LMG 20238^T^ (=DSM 16288^T^) of *Bacillus muralis* was isolated from mural paintings in the Lutheran church of Greene-Kreiensen, Germany (1). It is closely related to *Bacillus simplex* (1). The strains of the *B. simplex*/*B. muralis* group can be found frequently in deteriorated wall paintings (1, 2). Except for the taxonomic literature, no additional information of *B. muralis* has be obtained so far. Because of no available genomic information of *B. muralis*, its type strain LMG 20238^T^ was selected as one of the research objects in our genome sequencing project for genomic taxonomy and phylogenomics of *Bacillus*-like bacteria. Here, we present the first draft genome sequence of *B. muralis*.

The genome sequence of *B. muralis* LMG 20238^T^ was obtained by paired-end sequencing on the Illumina HiSeq 2500 system. One DNA library with an insert size of 275 bp was constructed and sequenced. After filtering of the 1.18-Gb raw data, the 1.12-Gb clean data were obtained, providing approximately 228-fold coverage. The reads were assembled via SOAPdenovo software version 1.05 (3), using a key parameter K setting at 68. Through the data assembly, 68 scaffolds with a total length of 5,183,886 bp were obtained, and the scaffold *N*_50_ was 227,643 bp. The average length of the scaffolds was 76,233 bp, and the longest and shortest scaffolds were 602,085 bp and 539 bp, respectively. A total of 95.12% clean reads could be aligned back to the genome, which covered 98.87% f the sequence.

The annotation of the genome was performed using the NCBI Prokaryotic Genomes Automatic Annotation Pipeline (PGAAP) (http://www.ncbi.nlm.nih.gov/books/NBK174280) utilizing GeneMark, Glimmer, and tRNAscan-SE tools (4). A total of 5,392 genes were predicted, including 5,261 coding sequences, 63 tRNAs, and 5 rRNAs. There were 3,624 and 2,268 genes assigned to the COG and KEGG databases, respectively. The average DNA G+C content was 40.95%, agreeing with the value 41.2 mol% (1).

### Nucleotide sequence accession numbers.

This whole-genome shotgun project has been deposited at DDBJ/EMBL/GenBank under the accession number LMBV00000000. The version described in this paper is the first version, LMBV01000000.
